# Bibliometric Analysis of Research on Vitamin D Deficiency and Its Association with Mental Health Outcomes Among Females

**DOI:** 10.3390/nu18142268

**Published:** 2026-07-11

**Authors:** Priyanka Patel, Priyadharshini Babu, Ravi Prakash Jha, Sonu Goel, Mitasha Singh, Soumen Barik, Mayank Singh

**Affiliations:** 1 Newcomb Institute, Tulane University, New Orleans, LA 70118, USA; 2 Centre for Disaster Mitigation and Management, Vellore Institute of Technology, Vellore 632014, India; 3 Department of Community Medicine, Dr. Baba Saheb Ambedkar Medical College and Hospital, Delhi 110085, India; 4 Department of Community Medicine, School of Public Health, Post Graduate Institute of Medical Education and Research, Chandigarh 160012, India; 5 Department of Fertility & Social Demography, International Institute for Population Sciences, Mumbai 400088, India; 6 Department of Epidemiology and Biostatistics, KLE Academy of Higher Education & Research, Belagavi 590010, India

**Keywords:** vitamin D deficiency, mental health outcomes, women’s health, bibliometric analysis, depression, anxiety, neurodegenerative diseases

## Abstract

Objective: The objective of this study is to analyze trends in global research on vitamin D deficiency and mental health outcomes among women using bibliometric methods, focusing on publication output, citation patterns, collaboration structures, thematic research clusters, and key gaps in the existing literature. Methods: A structured bibliometric search of the Scopus database identified 1124 English-language, peer-reviewed articles published between 2000 and 2024 on vitamin D deficiency and mental health in women. Data were analyzed using VOSviewer-Version 1.6.20 to visualize publication trends, citation impact, co-authorship networks, and thematic clusters. Basic statistics were used to explain the publication patterns, while the analysis of networks revealed new research themes and connections across different areas of study. Results: Research output increased steadily, reaching a peak in 2024. Annweiler, C. was the most prolific author. Leading institutions included the University of Western Ontario, Harvard T.H. Chan School of Public Health, and Monash University based on citation impact. The United States contributed the most publications, followed by the United Kingdom and China. Thematic analysis showed strong links between vitamin D deficiency and depression, anxiety, neurodegenerative disorders, and metabolic conditions. Conclusions: This bibliometric analysis highlights the growing academic interest in vitamin D deficiency and its association with mental health outcomes among women. The findings reflect patterns in research activity, collaboration, and thematic focus rather than evidence of clinical effectiveness or causal relationships. While significant progress has been made, gaps remain in gender-specific research, life stage analysis, and representation from low- and middle-income countries.

## 1. Introduction

Vitamin D deficiency, which impacts approximately 1 billion individuals globally, with a prevalence varying from 30% to 80% depending on the population, is a significant global health issue [1]. In India, research estimates that between 70 and 90% of the population can be vitamin D-deficient, and this deficiency is therefore severe both internationally and nationally. Depression and anxiety are significant determinants of the global burden of disease and have a greater impact on women. Depression is almost twice as common in women compared to men and represents a major public health burden [2].

Vitamin D is an important nutrient in human health, playing a key role in immune function, neurological processes, and mental well-being [1]. Gender is an important social determinant of health, and women are disproportionately affected by mental disorders. Emerging evidence suggests a potential association between vitamin D deficiency and mental health outcomes, particularly depression among women [3]. Vitamin D deficiency has been examined in relation to various mental health outcomes, including depression, anxiety, and mood-related conditions, although the findings remain inconsistent [4]. Although observational data indicate an association between vitamin D deficiency and the prevalence of depression, evidence from randomized controlled trials remains inconsistent. Some systematic reviews have reported no significant effect of vitamin D supplementation on mental health outcomes among healthy adults [5]. Meanwhile, meta-analyses of randomized controlled trials suggest a potential benefit in reducing depressive symptoms among individuals with clinically diagnosed depression [6]. Bibliometric analysis is a quantitative approach used to assess the scientific literature, providing useful information on trends in publication, top contributors, and thematic research areas [7].

The purpose of this study is to investigate the trend in global research on the lack of vitamin D and its association with mental health outcomes among women using a bibliometric approach. It uses bibliometric techniques, which means it analyzes data like how many times the research is cited. Specifically, this study examines publication output, citation patterns, and collaboration structures over the past 25 years and identifies major contributors, such as key authors, institutions, and countries involved in this research area. This research also compares highly cited papers, following their impact over time. Further, it evaluates thematic clusters in vitamin D and mental health research based on co-citation and keyword co-occurrence networks to determine emerging trends. By providing a structured bibliometric overview of the existing literature, this study identifies key knowledge gaps in women-focused research on vitamin D deficiency and mental health. In particular, it highlights the limited availability of comparative studies that examine sex differences between women and men, a gap that cannot be addressed directly within the scope of the present analysis but warrants further empirical investigation.

## 2. Materials and Methods

This study employed a bibliometric approach to analyze research trends, collaboration patterns, and thematic developments in studies on vitamin D deficiency and mental health outcomes among women. This bibliometric analysis examined research trends, collaboration patterns, and thematic evolution in studies on vitamin D deficiency and its association with mental health outcomes among women. Bibliometric data were retrieved from the Scopus database, which was selected due to its extensive multidisciplinary coverage, comprehensive citation indexing, and suitability for bibliometric analyses. Scopus was used as the sole data source to ensure consistency in citation metrics and metadata structure, in line with established bibliometric studies; however, this choice may limit the coverage of studies indexed exclusively in other databases. The literature search was conducted on 26 February 2025 using a structured Boolean query applied to the title, abstract, and author keywords (TITLE-ABS-KEY) fields. The complete search strategy is reported in Appendix A Table A1 to ensure reproducibility.

The search was restricted to peer-reviewed journal articles published in English between 2000 and 2024 that examined vitamin D deficiency and mental health outcomes among women. Clinical trials and observational studies were included, while reviews, editorials, conference papers, animal studies, case reports, self-reported studies, and non-journal sources were excluded. Metadata, including titles, abstracts, keywords, authors, affiliations, and citation counts, were exported in RIS format for further analysis.

The analysis was conducted using VOSviewer, focusing on key bibliometric indicators to assess publication output, citation trends, and keyword co-occurrence networks [7]. This approach helped identify dominant themes, research hotspots, and the evolution of research focus over time. In addition, a descriptive keyword-based stratification was conducted to explore variations in population focus and age-related themes within the dataset. Publication trends over time, country- and journal-level contributions, author productivity and citation impact, and collaboration patterns across authors, institutions, and countries were examined. Authorship and institutional contributions were analyzed to determine the productivity and impact of researchers and organizations. Co-authorship and institutional collaboration networks were visualized to map interactions between authors, institutions, and countries, highlighting influential researchers and key research hubs. Co-word analysis identified relationships between frequently occurring keywords, while cluster analysis grouped related keywords into thematic clusters, illustrating emerging trends in vitamin D deficiency and mental health research.

Duplicate records were evaluated using DOI and title screening. A random subset of publications was manually reviewed to confirm relevance and classification accuracy. Visual outputs such as keyword co-occurrence maps, collaboration networks, and trend graphs were generated using VOSviewer Version 1.6.20 to ensure clarity and interpretability. As this study utilized publicly available bibliometric data, ethical approval, consent to participate, and consent to publish were not required. The findings provide a comprehensive overview of research trends, collaboration networks, and thematic clusters in studies addressing vitamin D deficiency and mental health outcomes among women, offering insights for future research directions and identifying existing gaps in the literature.

**Figure 1:**
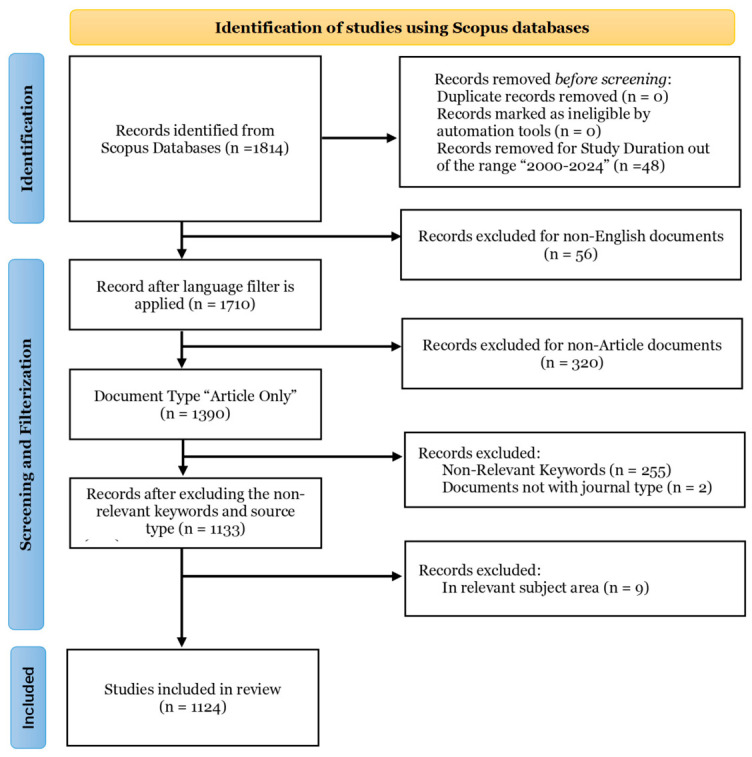
PRISMA flow diagram.

Figure 1 presents a bibliometric framework focusing on the search strategy, filtering criteria, and document selection process for analyzing the relationship between vitamin D deficiency, mental health disorders, and women’s health. Although a PRISMA-style flow diagram is used to enhance transparency in reporting the document selection process, this study does not constitute a systematic review. The PRISMA framework outlines a structured bibliometric approach to collecting, refining, and analyzing data from a vast number of documents to derive meaningful insights. The process begins with a structured Scopus search query combining vitamin D-related terms, mental health conditions, and female population keywords, as detailed in Appendix A Table A1. The initial search yielded 1814 documents, which were systematically filtered based on several criteria (Appendix A Table A1). Prior to screening, 48 records published outside the predefined publication period were removed. No duplicate records were identified. During screening, 56 non-English records and 320 non-article document types were excluded, resulting in 1390 records assessed for eligibility. Further refinement involved eliminating animal-related studies, case reports, and self-reported studies, which excluded 255 documents, leaving 1135 relevant records. Additional filtering by source type (journals only) and subject area (excluding engineering, chemistry, and computer science) resulted in a final dataset of 1124 high-quality, peer-reviewed documents.

The selected documents were then subjected to bibliometric indicators and mapping analysis, categorized into four key areas: Trend Analysis, Country and Journal Contribution, Author Contribution, and Co-Occurrence Analysis. Trend Analysis examined publication trends over time, identifying the growth of research in this domain. Country and Journal Contribution assessed which nations and journals contributed the most to the literature. Author Contribution identified the most cited works, highlighting key influential studies. Finally, Co-Occurrence Analysis explored the relationships between major keywords, uncovering prevalent research themes and interdisciplinary linkages. This comprehensive bibliometric approach ensures a structured, data-driven analysis, providing valuable insights into the evolving research landscape of vitamin D deficiency, mental health disorders, and women’s health.

## 3. Results

### 3.1. Bibliometric Overview of Publication Trends and Global Research Output

Table 1 presents a comprehensive bibliometric summary of 1124 journal articles published between 2000 and 2024, drawn from 609 distinct sources. The field demonstrates a robust annual growth rate of 16.89%, indicating rapidly expanding scholarly interest. The publications have an average document age of 6.52 years and receive a mean of 27.3 citations per article, reflecting moderate to high academic impact. Collectively, these studies cite 42,608 references, highlighting a well-established and extensively connected knowledge base. In terms of content, the dataset includes 7177 Keywords Plus (ID) and 2061 author-provided keywords (DE), suggesting substantial thematic diversity and methodological breadth. Authorship analysis shows contributions from 6785 authors, with only 26 authors producing single-authored documents, underscoring the strongly collaborative nature of research in this domain. Collaboration metrics further reinforce this pattern, with an average of 7.07 co-authors per document and 22.06% international co-authorship, indicating meaningful cross-national research engagement. All included documents are classified as original research articles, reflecting a consistent focus on empirical scholarly contributions.

**Figure 2:**
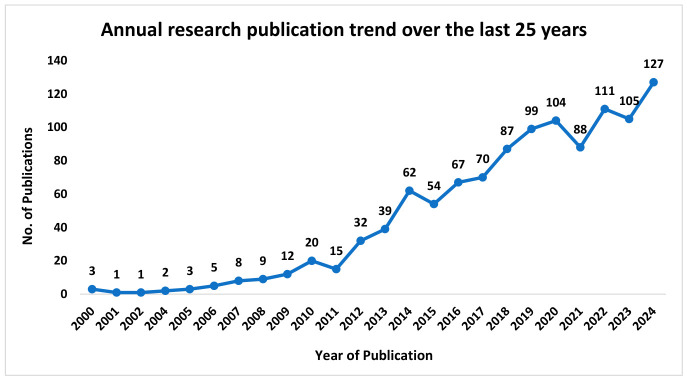
Annual trends in scientific publications on vitamin D deficiency and mental health outcomes (2000–2024).

The study analyzed annual research publication trends over a 25-year period, resulting in a marked and steadily increasing trajectory in research output (Figure 2). Publication counts were very low and sporadic during the early years (2000–2008), remaining below 10 articles per year. A clear upward shift is observed from 2010 onwards (*n* = 20), after which publications increased consistently, indicating growing scholarly interest in the field. The output rose sharply during the post-2015 period, crossing 50 publications by 2014 (*n* = 62) and exceeding 100 publications annually after 2019. The highest number of publications was recorded in 2024 (*n* = 127), representing the peak of research activity. Contrary to a decline, 2023 (*n* = 105) shows a slight recovery following a dip in 2022 (*n* = 88), suggesting short-term fluctuations rather than a sustained decrease.

**Figure 3:**
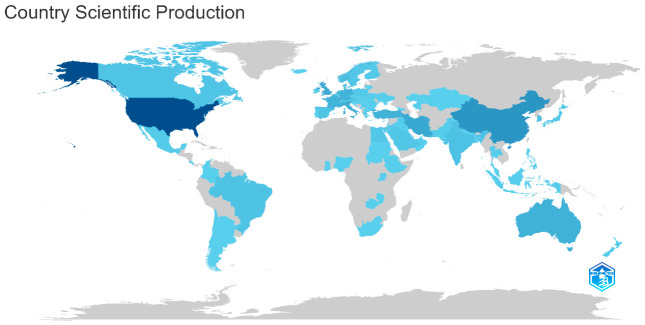
Global distribution of scientific publications on vitamin D deficiency and mental health outcomes. Darker shades indicate higher numbers of publications, while lighter shades represent lower publication counts.

Figure 3 illustrates the global distribution of scientific publications on vitamin D deficiency and mental health outcomes across countries. The United States emerges as the leading contributor, with the highest number of publications (*n* = 1526), followed by China (*n* = 649). The United Kingdom (*n* = 446) and Iran (*n* = 440) rank third and fourth, respectively. Other major contributors include Italy (*n* = 387), Australia (*n* = 336), Turkey (*n* = 332), Germany (*n* = 330), France (*n* = 326), and the Netherlands (*n* = 300). Overall, the spatial distribution highlights a strong concentration of research output in high-income and upper-middle-income countries, with the United States alone accounting for nearly 30% of publications among the top ten contributing nations.

### 3.2. Citation Impact and Scholarly Influence Across Authors, Countries, Journals, and Key Publications

The citation analysis revealed a substantial concentration of influence among a small group of highly cited authors (Table 2). The most cited author was Annweiler, C., with 1611 citations across 22 publications, followed by Vellas, B. (1480 citations; 8 documents) and Beauchet, O. (1374 citations; 17 documents). Notably, several authors (including Andreasen, N.; Boada, M.; Borrie, M.; and Bullock, R.) recorded an identical citation count of 784, despite contributing only one document each. At the institutional level, the University of Western Ontario, Canada, ranked the highest with 299 citations from three documents, followed by Harvard T.H. Chan School of Public Health, USA (206 citations; five documents), and Monash University, Australia (224 citations; three documents), indicating the strong institutional clustering of high-impact research.

Table 3 presents the leading countries and journals based on total citations and the number of documents published in the field of vitamin D deficiency and mental health outcomes. At the country level, the United States dominates scientific output, contributing the highest number of documents (*n* = 293) and accumulating 11,500 citations, indicating both high productivity and strong scholarly influence. This is followed by the United Kingdom (117 documents; 7080 citations) and France (57 documents; 4096 citations). Other notable contributors include Australia (73 documents; 3818 citations), Italy (59 documents; 3214 citations), Canada (49 documents; 2414 citations), and the Netherlands (59 documents; 2330 citations), reflecting broad engagement from high-income countries. In terms of journal sources, *Neurology* emerged as the most highly cited journal (1085 citations from six documents), highlighting its influence despite relatively fewer publications. *PLOS One* showed the highest productivity among journals, publishing 24 documents and receiving 811 citations. High-impact general and specialty journals such as the *New England Journal of Medicine* (784 citations from a single article), *Journal of Alzheimer’s Disease* (773 citations; 14 documents), and *Journals of Gerontology Series A* (710 citations; 8 documents) also contributed substantially to citation impact.

Table 4 presents the top 20 most highly cited documents published between 2004 and 2020, with total citation counts ranging from 784 to 191 as of 2025. The most cited article was by Honig et al. (2018), reporting a randomized trial of solanezumab for mild dementia due to Alzheimer’s disease, which accumulated 784 citations and the highest average annual citation rate (112 citations per year). This was followed by Gale et al.’s (2008) study on maternal vitamin D status during pregnancy (579 citations; 34 citations per year) and Bodnar et al. (2007) examining vitamin D insufficiency in pregnant women and neonates (562 citations; 31 citations per year). Several influential publications focused on cognitive decline, dementia, and neurodegenerative outcomes, including studies by Wilkins (2006), Llewellyn (2010), Jorde (2008), and Evatt (2008), each receiving over 300 citations, highlighting sustained scholarly attention to the vitamin D–cognition pathway. In addition, high-impact consensus and policy-oriented work, such as that by Conway et al. (2014), demonstrated strong visibility with 547 citations and a high annual citation rate (50 per year). More recent contributions, including that by Bischoff-Ferrari et al. (2020), achieved comparatively high average annual citation rates (47 per year) despite shorter publication windows, indicating rapid uptake and relevance.

### 3.3. Thematic Structure and Keyword Co-Occurrence Analysis

**Figure 4:**
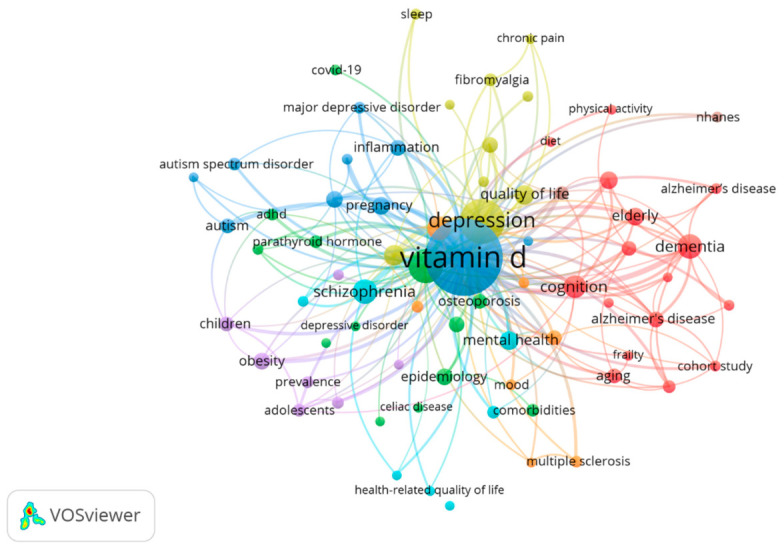
Co-occurrence network of author keywords in research on vitamin D deficiency and mental health outcomes.

Figure 4 shows the co-occurrence network of author keywords related to vitamin D and mental health-related themes. The central keyword, “vitamin D,” exhibited strong linkages with multiple terms associated with mental health and cognitive function. Prominent keywords directly related to mental health include “depression,” “major depressive disorder,” “mental health,” “mood,” “schizophrenia,” “ADHD,” “autism,” “autism spectrum disorder,” and “depressive disorder.” In addition, a significant cluster focused on cognitive and ageing-related conditions, featuring terms such as “dementia,” “Alzheimer’s disease,” “cognition,” and “elderly.”

**Figure 5:**
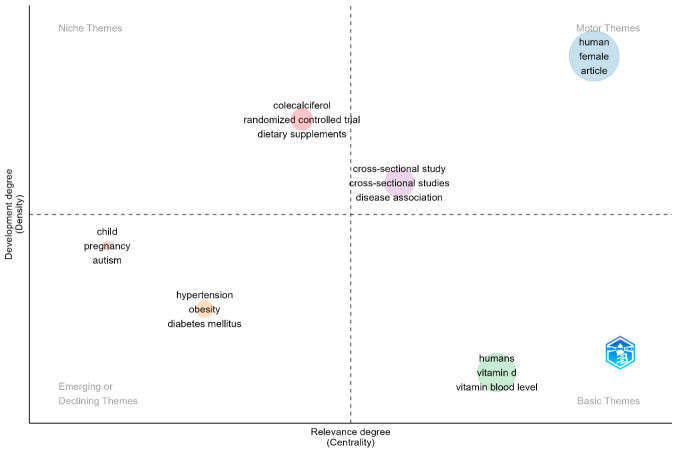
Thematic mapping of author keywords in vitamin D deficiency and mental health research.

A thematic map of author keywords in research on vitamin D and metal health outcomes (Figure 5) shows that motor themes such as human and female are well-developed and central to the field. Basic themes, including vitamin D and its blood levels and the role of humans, are foundational but underdeveloped. Niche themes like colecalciferol, randomized controlled trials, and dietary supplements are specialized but less central. Emerging or declining themes such as child, pregnancy, autism, hypertension, obesity, and diabetes mellitus reflect areas of limited development and relevance. The map outlines both core and peripheral directions in current research.

### 3.4. Co-Citation Networks and Influential Research Clusters

**Figure 6:**
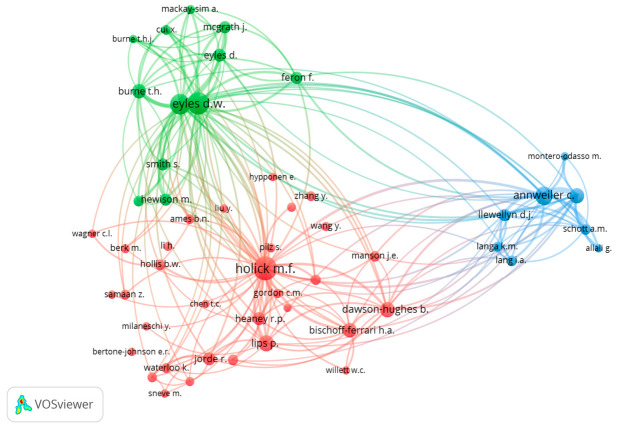
Co-citation network visualization of influential publications in vitamin D deficiency and mental health research.

The network visualization of co-citation analysis based on author keywords (Figure 6) shows the three significant clusters of highly co-cited writers in the field of vitamin D research. The red cluster, led by Holick M.F., includes influential authors such as Pilz S., Heaney R.P., Bischoff-Ferrari H.A., and Gordon C.M., reflecting strong collaboration in clinical and supplementation-related studies. The blue cluster focuses on research related to cognitive decline, ageing, and dementia outcomes; is led by Annweiler C.; and includes researchers such as Llewellyn D.J., Schott A.M., and Montero-Odasso M. In contrast, the green cluster centres around Eyles D.W., followed by McGrath J., Burne T.H., and Feron F., reflecting strong interest in the neurodevelopmental and mechanistic aspects of vitamin D.

## 4. Discussion

This study maps the evolving global research landscape on vitamin D deficiency and its association with mental health outcomes, with a focus on females. An analysis of 1124 original research articles published between 2000 and 2024 reveals a field in rapid expansion, marked by a 16.89% annual growth rate and a steep rise in output since 2010, peaking at 127 publications in 2024. The literature demonstrates moderate-to-high academic impact (mean 27.3 citations per article), a well-integrated knowledge base (42,608 cited references), and a deeply collaborative structure (7.07 co-authors per article; 22.06% international co-authorship). These patterns confirm that research at this intersection has matured into a sustained, interdisciplinary, and empirically driven scientific domain, increasingly centring on research examining potential associations between vitamin D status and mental health with emerging, though still limited, attention to female populations [2,8]. It is important to emphasize that the findings of this study reflect patterns in scientific research activity and publication trends rather than evidence of clinical effectiveness or causal relationships between vitamin D deficiency and mental health outcomes. The observed thematic clusters indicate areas of research focus and scientific interest rather than confirmed biological or clinical effects.

### 4.1. Influential Contributors and Collaboration Patterns

Our analysis highlights a highly collaborative research landscape, with influential contributions from key authors such as Annweiler, C.; Vellas, B.; and Beauchet, O., particularly in the domain of geriatric and neurocognitive health. The prominence of institutions such as the University of Western Ontario (Canada), Monash University (Australia), and Harvard T.H. Chan School of Public Health (USA) reflects the concentration of high-impact research (on citation basis) within well-established academic networks that benefit from strong research infrastructure and international collaborations. Furthermore, the global distribution of research output is predominantly driven by high-income countries, including the United States, the United Kingdom, France, and Australia. However, there is some meaningful research contribution from upper-middle-income countries such as Iran (*n* = 440) and Turkey (*n* = 332), with limited representation from low-income settings. This imbalance suggests a disconnect between regions with a high burden of vitamin D deficiency among women and those contributing the most to the scientific literature. Strengthening international research collaborations and capacity-building initiatives in low- and middle-income countries is essential to address this gap and generate more context-specific evidence to inform public health strategies [1,5,9].

### 4.2. Geographic Concentration and Journal Influence

Research is heavily concentrated in high-income countries, with the United States leading in both output (293 documents) and influence (11,500 citations), being nearly double that of the next most productive nation. The United Kingdom, France, Australia, Italy, Canada, and the Netherlands further reinforce a global knowledge base shaped predominantly by Western academic ecosystems. This geographic skew limits insight into populations with high vitamin D deficiency burdens but low research capacity, particularly in low- and middle-income regions [10]. In terms of scholarly venues, impact is driven less by volume and more by placement in high-visibility journals. *Neurology*, with only six articles, garnered 1085 citations, underscoring the centrality of neurological and cognitive outcomes in this field [11]. *PLOS One* leads in productivity (24 articles) but with moderate per-paper impact, reflecting its broad research scope and role as a platform for wide dissemination [12]. Landmark contributions in *NEJM* (784 citations from one article), *Journal of Alzheimer’s Disease*, and *Journals of Gerontology Series A* confirm that the most influential work clusters around neurodegeneration, ageing, and clinical trial evidence, further marginalizing non-cognitive mental health dimensions, especially in younger or non-pregnant females.

### 4.3. Major Research Trends

Our analysis highlighted a constant increase in research publications from 2000 to 2024, reaching a peak in 2024 with 127 publications. This trend reflects the growing academic recognition of vitamin D deficiency as a prominent research topic of interest in relation to mental health outcomes among females. A slight dip in output was observed in 2022 (*n* = 88), followed by a recovery in 2023 (*n* = 105), rather than a sustained decline; this fluctuation may be partially attributable to shifts in global research priorities toward the COVID-19 pandemic and infectious disease management, including vaccine development [13]. However, the upward trajectory reflected continuous academic engagement with vitamin D deficiency and multidimensional women’s health impacts.

Severe mental health conditions, such as depression, major depressive disorder, schizophrenia, ADHD, and autism spectrum disorder, as well as cognitive decline and neurodegenerative disorders including Alzheimer’s disease and dementia, emerged as prominent research themes in the keyword co-occurrence analysis [6,14]. These results are consistent with the global burden of mental health conditions, which women are affected by disproportionally, being twice as prevalent in women as compared to men [15]. Our analysis highlights these conditions as prominent research themes within the literature [16,17]. Also, there is growing academic interest in finding the link between vitamin D deficiency and neurodegenerative conditions in older women, such as Alzheimer’s disease and dementia [18].

### 4.4. Highly Cited Studies and Their Impact

The field is anchored by a few high-impact studies, including some indirectly related to vitamin D but being central through co-citation. Honig et al.’s (2018) trial on Alzheimer’s therapeutics leads in citation counts (784), reflecting the strong integration of vitamin D research within the dementia literature. More directly relevant are [19,20], whose work on maternal and neonatal vitamin D deficiency remains highly influential (579 and 562 citations, respectively), positioning pregnancy as a key focus in female-related research. Seminal contributions by [21], Jorde (2008), and Evatt (2008) solidified the vitamin D–cognition link in ageing populations, while consensus papers like that by Conway (2014), with 547 citations, shaped clinical discourse. Reference [9] with a high annual citation rate (47) signals growing interest in intervention science. Notably, despite the emphasis on females in perinatal and geriatric contexts, few top-cited studies examine the relationship between vitamin D deficiency and mood, anxiety, or neurodevelopmental disorders across the broader female lifespan—revealing a critical gap between citation influence and comprehensive gender-focused inquiry [22].

### 4.5. Thematic Clusters and Emerging Trends

In keyword co-occurrence and co-citation analysis, we found unique themes in the green clusters, which include neurodegenerative diseases, mental health issues and metabolic disorders. Mental health conditions such as ADHD, schizophrenia, and autism are prominent themes within the literature, alongside research examining the relationship between vitamin D status and neurodevelopmental and psychiatric disorders [23]. Ageing, dementia, and Alzheimer’s disease were highlighted in the red cluster, emphasizing its relevance to the association of neurodegenerative conditions [24,25]. The blue cluster captures broader risk factors, including ageing-related and nutritional dimensions relevant to overall health. These clusters illustrate the multidisciplinary nature of vitamin D research and its broad implications for women’s health [26]. Supplementation (red), cognitive health (blue), and neurodevelopment (green) are the three main research directions we found regarding vitamin D from the co-citation network. The neurodevelopment cluster (green) is comparatively smaller, indicating an emerging area of research, whereas the supplementation and cognitive health clusters are more established. Limited overlap between clusters suggests fragmented research. Further studies should strengthen interdisciplinary links, particularly examining the relationship between vitamin D status and brain development in women’s mental health. Stronger collaboration between these research domains may enhance the future understanding of how vitamin D is studied in relation to women’s mental health across different life stages.

## 5. Limitations

This study has several limitations. First, the analysis was restricted to the Scopus database and English-language publications, which may have excluded relevant studies indexed in other databases such as PubMed or Web of Science. Second, bibliometric methods rely on citation data, which may favour older or highly cited publications and may not fully reflect recent research developments. Third, variability in keyword usage across studies may have influenced the identification of thematic clusters.

Additionally, the use of broad mental health search terms limited the ability to distinguish specific conditions, such as postpartum depression or life-stage-specific outcomes. Importantly, this study is descriptive in nature and did not assess clinical effectiveness or causal relationships. The findings should therefore be interpreted as patterns in research activity. This study did not differentiate between studies reporting positive or negative outcomes, as the bibliometric approach focused on publication patterns and thematic structures rather than effect direction.

## 6. Implications of This Study

This study highlights key research trends and gaps in the literature on vitamin D deficiency and mental health among women. The findings indicate growing scientific interest but also reveal imbalances in geographic representation and limited focus on specific life stages and populations. Future research should priorities more targeted investigations, particularly among underrepresented populations and low- and middle-income countries. Strengthening interdisciplinary collaboration and improving data sharing across regions may enhance the development of this research area. While this study identifies temporal trends in publication output, these patterns should be interpreted descriptively rather than as evidence of underlying causal relationships.

## 7. Conclusions

This bibliometric analysis provides a comprehensive overview of trends in global research on vitamin D deficiency and mental health outcomes among women. The findings demonstrate increasing scientific interest and identify key thematic areas, including depression, cognitive decline, and neurodevelopment. Importantly, these results reflect patterns in research activity rather than evidence of clinical effectiveness or causal relationships. Several gaps remain, including limited focus on women-specific populations, life stage variation, and the underrepresentation of low- and middle-income countries. Future research should address these gaps through more targeted, context-specific, and interdisciplinary approaches to better understand this evolving field.

## Figures and Tables

**Table 1:** Descriptive bibliometric summary of scientific publications (2000–2024).

Description	Results
**MAIN INFORMATION ABOUT DATA**	
Timespan	2000:2024
Sources (Journals, Books, etc.)	609
Documents	1124
Annual Growth Rate %	16.89
Document Average Age	6.52
Average Citations per Doc	27.3
References	42,608
**DOCUMENT CONTENTS**	
Keywords Plus (ID)	7177
Author Keywords (DE)	2061
AUTHORS	
Authors	6785
Authors of Single-Authored Docs	26
**AUTHORS COLLABORATION**	
Single-Authored Docs	27
Co-Authors per Doc	7.07
International Co-Authorships %	22.06
**DOCUMENT TYPES**	
article	1124

**Table 2:** Top authors and organizations according to highest number of citations and number of documents published.

Citations	Author	Documents	Citations	Organizations/Institutes	Documents
1611	Annweiler, C	22	299	Department Of Medical Biophysics, Schulich School of Medicine and Dentistry, University of Western Ontario, London, On, Canada	3
1480	Vellas, B	8	224	Department Of Epidemiology and Preventive Medicine, Monash University, Melbourne, Vic, Australia	3
1374	Beauchet, O	17	206	Department Of Epidemiology, Harvard T. H. Chan School of Public Health, Boston, Ma, United States	5
1241	Llewellyn, Dj	8	206	Department Of Rehabilitation and Geriatrics, Geneva University Hospitals, University of Geneva, Geneva, Switzerland	3
1004	Lang, Ia	5	163	Queensland Centre For Mental Health Research, The Park Centre for Mental Health, Wacol, Qld, Australia	3
1004	Langa, Km	5	123	National Centre for Register-Based Research, Aarhus University, Aarhus, Denmark	4
784	Andreasen, N	1	122	Department Of Nutrition, School of Public Health, Iran University of Medical Sciences, Tehran, Iran	5
784	Boada, M	1	117	Department Of Cellular and Molecular Nutrition, School of Nutritional Sciences and Dietetics, Tehran University of Medical Sciences, Tehran, Iran	4
784	Borrie, M	1	103	Department Of Biostatistics, University Of Washington, Seattle, Wa, United States	3
784	Bullock, R	1	74	Department Of Community Nutrition, School of Nutritional Sciences and Dietetics, Tehran University of Medical Sciences, Tehran, Iran	3
784	Carlson, C	1	69	Pediatric Growth and Development Research Center, Institute of Endocrinology and Metabolism, Iran University of Medical Sciences, Tehran, Iran	3
784	Case, M	1	67	Mrc Centre For Causal Analyses in Translational Epidemiology, School of Social and Community Medicine, University of Bristol, Bristol, United Kingdom	3
784	Dean, Ra	1	52	Department Of Internal Medicine and Clinical Pharmacology, Medical University of Silesia, Katowice, Poland	3
784	Demattos, R	1	46	Robarts Research Institute, Department of Medical Biophysics, Schulich School of Medicine and Dentistry, The University of Western Ontario, London, On, Canada	3
784	Hager, K	1	42	Harvard Medical School, Boston, Ma, United States	3
784	Hake, A	1	38	Research Center of Psychiatry and Behavioral Sciences, Tabriz University of Medical Sciences, Tabriz, Iran	3
784	Hoffmann, Vp	1	34	Department Of Clinical Nutrition, School of Nutrition and Food Science, Isfahan University of Medical Sciences, Isfahan, Iran	3
784	Honig, Ls	1	32	Department Of Epidemiology, Columbia University Mailman School of Public Health, New York, Ny, United States	3
784	Khanna, R	1	28	Department Of Clinical Pharmacy, Faculty of Pharmacy, Jordan University of Science and Technology, Irbid, Jordan	4
784	Liu-Seifert, H	1	28	Department Of Obstetrics and Gynecology, Cedars-Sinai Medical Center, Los Angeles, Ca, United States	3
784	Mintun, M	1			
784	Scarpini, E	1			
784	Selzler, Kj	1			
784	Siemers, E	1			
784	Sundell, K	1			
784	woodward, m	1			

**Table 3:** Top countries and journals according to highest number of citations and number of documents published.

Citations	Country	Documents	Citations	Source/Journal	Documents
11,500	United States	293	1085	*Neurology*	6
7080	United Kingdom	117	811	*PLOS One*	24
4096	France	57	784	*New England Journal of Medicine*	1
3818	Australia	73	773	*Journal of Alzheimer’s Disease*	14
3214	Italy	59	710	*Journals of Gerontology—Series A Biological Sciences and Medical Sciences*	8
2414	Canada	49	669	*European Journal of Endocrinology*	4
2330	Netherlands	59	648	*European Journal of Clinical Nutrition*	2
2226	Germany	55	626	*Nutrients*	50
2052	Spain	22	623	*Schizophrenia Research*	13
1739	Sweden	31	618	*Journal of Nutrition*	6
1705	China	97	507	*JAMA—Journal of The American Medical Association*	3
1528	Switzerland	26	444	*Nutritional Neuroscience*	15
1511	Turkey	71	437	*Archives of Internal Medicine*	2
1365	Iran	75	428	*Journal of The American Geriatrics Society*	6
1295	Norway	28	394	*American Journal of Geriatric Psychiatry*	2
907	Denmark	31	381	*Journal of Clinical Endocrinology and Metabolism*	6
850	Portugal	6	379	*Molecular Psychiatry*	3
757	Japan	20	375	*Journal of Nutrition, Health and Aging*	11
712	Austria	15	361	*Journal of Internal Medicine*	1
632	Greece	12	354	*Journal of Affective Disorders*	18

**Table 4:** Top 20 documents according to citations along with their average citations.

Author	Title	Citations	Year of Publication	Current Period	Average Citations Per Year
Honig (2018)	Trial of solanezumab for mild dementia due to Alzheimer’s disease	784	2018	2025	112
Gale (2008)	Maternal vitamin D status during pregnancy and child outcomes	579	2008	2025	34
Bodnar (2007)	High prevalence of vitamin D insufficiency in black and white pregnant women residing in the northern United States and their neonates	562	2007	2025	31
Conway (2014)	The polycystic ovary syndrome: A position statement from the European Society of Endocrinology	547	2014	2025	50
Littlejohns (2014)	Vitamin D and the risk of dementia and Alzheimer disease	418	2014	2025	38
Wilkins (2006)	Vitamin D deficiency is associated with low mood and worse cognitive performance in older adults	394	2006	2025	21
Llewellyn (2010)	Vitamin D and risk of cognitive decline in elderly persons	377	2010	2025	25
Jorde (2008)	Effects of vitamin D supplementation on symptoms of depression in overweight and obese subjects: Randomized double-blind trial	361	2008	2025	21
Evatt (2008)	Prevalence of vitamin D insufficiency in patients with Parkinson disease and Alzheimer disease	320	2008	2025	19
Buell (2010)	25-Hydroxyvitamin D, dementia, and cerebrovascular pathology in elders receiving home services	276	2010	2025	18
Mcgrath (2004)	Vitamin D supplementation during the first year of life and risk of schizophrenia: A Finnish birth cohort study	267	2004	2025	13
Han (2011)	Obesity and weight management in the elderly	250	2011	2025	18
Bischoff-Ferrari (2020)	Effect of Vitamin D Supplementation, Omega-3 Fatty Acid Supplementation, or a Strength-Training Exercise Program on Clinical Outcomes in Older Adults: The DO-HEALTH	234	2020	2025	47
Milaneschi (2014)	The association between low vitamin D and depressive disorders	203	2014	2025	18
Peterli (2017)	Laparoscopic sleeve gastrectomy versus Roux-Y-Gastric bypass for morbid obesity—3-year outcomes of the prospective randomized Swiss Multicenter Bypass Or Sleeve Study (SM-BOSS)	201	2017	2025	25
Masoumi (2009)	1α,25-dihydroxyvitamin D3 interacts with curcuminoids to stimulate amyloid-β clearance by macrophages of alzheimer’s disease patients	201	2009	2025	13
Holmes (2009)	Vitamin D deficiency and insufficiency in pregnant women: A longitudinal study	200	2009	2025	13
Przybelski (2007)	Is vitamin D important for preserving cognition? A positive correlation of serum 25-hydroxyvitamin D concentration with cognitive function	194	2007	2025	11
Milaneschi (2010)	Serum 25-hydroxyvitamin D and depressive symptoms in older women and men	192	2010	2025	13
Armstrong (2007)	Vitamin D deficiency is associated with anxiety and depression in fibromyalgia	191	2007	2025	11

## Data Availability

The data used in this study were obtained from the Scopus database and consist of bibliographic records (titles, abstracts, keywords, citations, and metadata) of published articles. These data are publicly accessible to authorized users through Scopus (Elsevier). No primary data were generated for this study. Data extraction and analysis were conducted in accordance with the database’s terms of use.

## Appendix A

**Table A1:** Search strategy, filtering criteria, and inclusion/exclusion of documents.

Criteria	Excluded Documents	Included Documents
**Search Date:** 26 February 2025		**1814**
**Database:** Scopus		
**Initial Search Term:** (TITLE-ABS-KEY (“Vitamin D deficiency” OR “Vitamin D Deficiencies” OR “Vitamin D insufficiency” OR “Hypovitaminosis D” OR “low Vitamin D levels” OR “Cholecalciferol”)) AND (TITLE-ABS-KEY (“Depression” OR “Depressions” OR “Depressive Symptoms” OR “psychological distress” OR “suicidal ideation” OR “suicidality” OR “suicide attempt” OR “Mental Hygiene” OR “Mental Health” OR “Stress Disorder” OR “posttraumatic stress disorder” OR “Moral Injury” OR “Moral Injuries” OR “alcohol abuse” OR “Schizophrenia” OR “Anxiety” OR “Bipolar Disorder” OR “Autism Spectrum Disorder” OR “Psychosis” OR “Dementia” OR “Cognitive Decline” OR “Seasonal Affective Disorder” OR “Attention Deficit Hyperactivity Disorder” OR “Obsessive-Compulsive Disorder” OR “Alzheimer’s disease” OR “Alzheimer Syndrome” OR “Alzheimer”)) AND (TITLE-ABS-KEY (“Women” OR “women’s” OR “females” OR “female” OR “pregnant women” OR “postmenopausal women” OR “adolescent girls” OR “reproductive women” OR “Girl” OR “Girls”))		
**Year:** 2000–2024	**48**	**1766**
**Language:** English	**56**	**1710**
**Document Type:** Article	**320**	**1390**
**Excluded Keywords:** Animal, Animal model, Animal Experiment, Animals, Nonhuman, Case Report and Self Report	**255**	**1135**
**Source Type:** Journal	**2**	**1133**
**Subject Area Excluded:** Engineering, Chemistry, Chemical Engineering, Computer Science, Mathematics	**9**	**1124**
**Duplication removal** on the basis of “DOI and Title screening”	**0**	**1124**

Note: This table outlines the systematic procedure followed to finalize a corpus of 1124 articles for review. The search terms were developed through a brainstorming session among the authors, which included subject matter and methodological experts.

## References

[1] Holick M.F. (2017). The vitamin D deficiency pandemic: Approaches for diagnosis, treatment and prevention. Rev. Endocr. Metab. Disord..

[2] Albert P.R. (2015). Why is depression more prevalent in women?. J. Psychiatry Neurosci..

[3] Moy F.M., Hoe V.C., Hairi N.N., Vethakkan S.R., Bulgiba A. (2017). Vitamin D deficiency and depression among women from an urban community in a tropical country. Public Health Nutr..

[4] Silva M.R.M., Barros W.M.A., da Silva M.L., da Silva J.M.L., Souza A.P.d.S., da Silva A.B.J., Fernandes M.S.d.S., de Souza S.L., Souza V.d.O.N. (2021). Relationship between vitamin D deficiency and psychophysiological variables: A systematic review of the literature. Clinics.

[5] Guzek D., Kołota A., Lachowicz K., Skolmowska D., Stachoń M., Głąbska D. (2021). Association between Vitamin D Supplementation and Mental Health in Healthy Adults: A Systematic Review. J. Clin. Med..

[6] Ghaemi S., Zeraattalab-Motlagh S., Jayedi A., Shab-Bidar S. (2024). The effect of vitamin D supplementation on depression: A systematic review and dose–response meta-analysis of randomized controlled trials. Psychol. Med..

[7] Donthu N., Kumar S., Mukherjee D., Pandey N., Lim W.M. (2021). How to conduct a bibliometric analysis: An overview and guidelines. J. Bus. Res..

[8] Menon V., Kar S.K., Suthar N., Nebhinani N. (2020). Vitamin D and depression: A critical appraisal of the evidence and future directions. Indian J. Psychol. Med..

[9] Bischoff-Ferrari H.A., Vellas B., Rizzoli R., Kressig R.W., Da Silva J.A., Blauth M., Felson D.T., McCloskey E.V., Watzl B., Hofbauer L.C. (2020). Effect of vitamin D supplementation, omega-3 fatty acid supplementation, or a strength-training exercise program on clinical outcomes in older adults: The DO-HEALTH randomized clinical trial. JAMA.

[10] Roth D.E., Abrams S.A., Aloia J., Bergeron G., Bourassa M.W., Brown K.H., Calvo M.S., Cashman K.D., Combs G., De-Regil L.M. (2018). Global prevalence and disease burden of vitamin D deficiency: A roadmap for action in low- and middle-income countries. Ann. N. Y. Acad. Sci..

[11] Littlejohns T.J., Henley W.E., Lang I.A., Annweiler C., Beauchet O., Chaves P.H., Fried L., Kestenbaum B.R., Kuller L.H., Langa K.M. (2014). Vitamin D and the risk of dementia and Alzheimer disease. Neurology.

[12] Dean A.J., Bellgrove M.A., Hall T., Phan W.M.J., Eyles D.W., Kvaskoff D., McGrath J.J. (2011). Effects of Vitamin D Supplementation on Cognitive and Emotional Functioning in Young Adults—A Randomised Controlled Trial. PLoS ONE.

[13] Singh P., Anand A., Rana S., Kumar A., Goel P., Kumar S., Gouda K.C., Singh H. (2024). Impact of COVID-19 vaccination: A global perspective. Front. Public Health.

[14] Akhtar S. (2016). Vitamin D Status in South Asian Populations—Risks and Opportunities. Crit. Rev. Food Sci. Nutr..

[15] Abate K. (2013). Gender Disparity In Prevalence of Depression Among Patient Population: A Systematic Review. Ethiop. J. Health Sci..

[16] Chai B., Gao F., Wu R., Dong T., Gu C., Lin Q., Zhang Y. (2019). Vitamin D deficiency as a risk factor for dementia and Alzheimer’s disease: An updated meta-analysis. BMC Neurol..

[17] Lauer A.A., Janitschke D., Hartmann T., Grimm H.S., Grimm M.O., Fedotova J. (2020). The Effects of Vitamin D Deficiency on Neurodegenerative Diseases. Vitamin D Deficiency.

[18] Khatoon R. (2025). Unlocking the Potential of Vitamin D: A Comprehensive Exploration of Its Role in Neurological Health and Diseases. Biology.

[19] Gale C.R., Robinson S.M., Harvey N.C., Javaid M.K., Jiang B., Martyn C.N., Godfrey K.M., Cooper C. (2008). Maternal vitamin D status during pregnancy and child outcomes. Eur. J. Clin. Nutr..

[20] Bodnar L.M., Simhan H.N., Powers R.W., Frank M.P., Cooperstein E., Roberts J.M. (2007). High Prevalence of Vitamin D Insufficiency in Black and White Pregnant Women Residing in the Northern United States and Their Neonates. J. Nutr..

[21] Llewellyn D.J., Lang I.A., Langa K.M., Muniz-Terrera G., Phillips C.L., Cherubini A., Ferrucci L., Melzer D. (2010). Vitamin D and risk of cognitive decline in elderly persons. Arch. Intern. Med..

[22] AlGhamdi S.A. (2024). Effectiveness of Vitamin D on Neurological and Mental Disorders. Diseases.

[23] Rihal V., Khan H., Kaur A., Singh T.G., Abdel-Daim M.M. (2022). Therapeutic and mechanistic intervention of vitamin D in neuropsychiatric disorders. Psychiatry Res..

[24] Berridge M.J. (2018). Vitamin D deficiency: Infertility and neurodevelopmental diseases (attention deficit hyperactivity disorder, autism, and schizophrenia). Am. J. Physiol.-Cell Physiol..

[25] Cuomo A., Maina G., Bolognesi S., Rosso G., Beccarini Crescenzi B., Zanobini F., Goracci A., Facchi E., Favaretto E., Baldini I. (2019). Prevalence and Correlates of Vitamin D Deficiency in a Sample of 290 Inpatients With Mental Illness. Front. Psychiatry..

[26] Neo B., Qu X., Dunlop E., Shepherd C., Walsh E.I., Cherbuin N., Black L.J. (2023). Mapping the citation network on vitamin D research in Australia: A data-driven approach. Front. Med..

